# Editorial for the Special Issue “Global Excellence in Bioactive Gels”

**DOI:** 10.3390/gels11060433

**Published:** 2025-06-05

**Authors:** Yoshitaka Miyamoto

**Affiliations:** 1 Department of Maternal-Fetal Biology, National Research Institute for Child Health and Development, Setagaya-ku, Tokyo 157-8535, Japan; myoshi1230@gmail.com or miyamoto-ys@ncchd.go.jp; Tel.: +81-3-3416-0181; 2 Graduate School of Engineering, Tokyo University of Agriculture and Technology, Koganei, Tokyo 184-8588, Japan; 3 Department of Mechanical Engineering, Institute of Science Tokyo, Meguro-ku, Tokyo 152-8552, Japan

This Special Issue provides an overview of “Global Excellence in Bioactive Gels”. Bioactive gels are used globally in medicine [1,2,3], drug discovery systems (DDSs) [4,5], cosmetics [6], food products [7,8], the environment [9], etc. Gels have a complex three-dimensional network structure and exhibit various physical properties. For example, hydrogels are natural or synthetic polymers that swell when exposed to water. This enables them to discontinuously and reversibly change their volume in response to external conditions such as the temperature, solvent, composition, light, and electric field. Extracellular matrix hydrogels (fibrous proteins such as collagen, and polysaccharides such as hyaluronic acid), synthetic polymer hydrogels, and rigid polymer materials have been used as scaffold materials; in chromatography; as membranes; and in drug delivery systems (DDSs), transplantation, regenerative therapy, biosensors, and biological fuel cells. For this Special Issue, after a comprehensive review process, eight high-quality works were accepted for publication.

Seven of these papers are original articles on bioactive gels. Yamagishi R et al. [10] studied a technology for producing self-dissolving hyaluronic acid gel ultrafine microneedles for medical applications. Mahamoud MM et al. [11] used tannic acid as a crosslinking agent, and by applying multiple freeze–thaw cycles, they enhanced the mechanical properties of hydrogels composed of polyvinyl alcohol (PVA) and agar. Zeng T et al. [12] demonstrated that the stiffness of the extracellular matrix (ECM) plays an important role in the development and progression of breast cancer. The authors prepared agarose hydrogels of varying stiffnesses that mimic the extracellular matrix (ECM) and investigated their effect on the chemotherapy resistance of breast cancer cells. Tomasello L et al. [13] show that amine-functionalized gellan-gum-based hydrogels containing adipose-stem-cell-derived small extracellular vesicles are effective in inflammatory environments against human dermal fibroblasts. Cuc S et al. [14] evaluated the teeth-whitening effects of gels containing papain and bromelain and compared their ability to remove stains caused by coffee and natural fruit juice. Alves RO et al. [15] used bovine teeth to evaluate the effect of quercetin-doped hydrogen peroxide gels on enamel properties. Bjelošević Žiberna M et al. [16] demonstrated the potential of using whey, a byproduct of dairy products, to develop sustainable cosmetic products (cleansing hydrogel and shampoo). One of the review articles in this Special Issue is on bioactive gels. Agbna GHD et al. [17] summarize the performance of hydrogels and their application in boosting plants’ resilience to water stress for sustainable agriculture.

In conclusion, this Special Issue, “Global Excellence in Bioactive Gels”, presents manuscripts that evaluate the potential of bioactive gels for application in medicine, drug discovery systems (DDSs), cosmetics, and the environment.

Dr. Yoshitaka Miyamoto would like to thank the Editor-in-Chief, Prof. Dr. Esmaiel Jabbari, as well as the editorial team and the reviewers of *Gels*, who helped us to publish this Special Issue.

Many of the papers included in this Special Issue are from the medical and materials fields, including the field of bioactive gels. Therefore, we look forward to receiving submissions from more diverse fields for the next Special Issue, “Global Excellence in Bioactive Gels (Second Edition)”.
